# Clofazimine Enteropathy: A Rare and Underrecognized Complication of Mycobacterial Therapy

**DOI:** 10.1093/ofid/ofw004

**Published:** 2016-09-02

**Authors:** Winnie Szeto, Monica T. Garcia-Buitrago, Lilian Abbo, Joseph D. Rosenblatt, Baharak Moshiree, Michele I. Morris

**Affiliations:** 1 Divisions of Gastroenterology; 2 Division of Pathology; 3 Infectious Diseases; 4 Division of Hematology and Oncology, Department of Medicine, University of Miami, Leonard M. Miller School of Medicine, Florida

**Keywords:** clofazimine, clofazimine-induced enteropathy, crystal storing histiocytosis enteropathy, *Mycobacterium abscessus*, petechial hemorrhage

## Abstract

Clofazimine-induced crystal-storing histiocytosis is a rare complication of treatment previously reported in dermatology literature as a complication of leprosy therapy. We report a case of disseminated *Mycobacterium abscessus* requiring treatment with high-dose oral clofazimine resulting in enteropathy in a patient who presented with abdominal pain, malnutrition, and melena.

Clofazimine is an antimicrobial that has been in clinical use for almost 40 years, primarily for the treatment of multibacillary leprosy. It binds to the guanine bases of bacterial DNA, blocking its function and inhibiting bacterial proliferation. It also increases activity of bacterial phospholipase A2, leading to release and accumulation of lysophospholipids thus inhibiting bacterial proliferation [1, 2]. With the increased prevalence of pulmonary and extrapulmonary nontuberculous mycobacterial disease as well as the emergence of multidrug-resistant (MDR) tuberculosis, clofazimine has acquired new prominence as a therapeutic agent [3]. In this report, we present a case of clofazimine-induced enteropathy in an immunocompromised patient on high-dose clofazimine for treatment of disseminated *Mycobacterium abscessus* to highlight the characteristic clinical history and pathologic findings present in the gastrointestinal tract in patients with clofazimine-induced enteropathy.

## CASE REPORT

**Figure 1.**
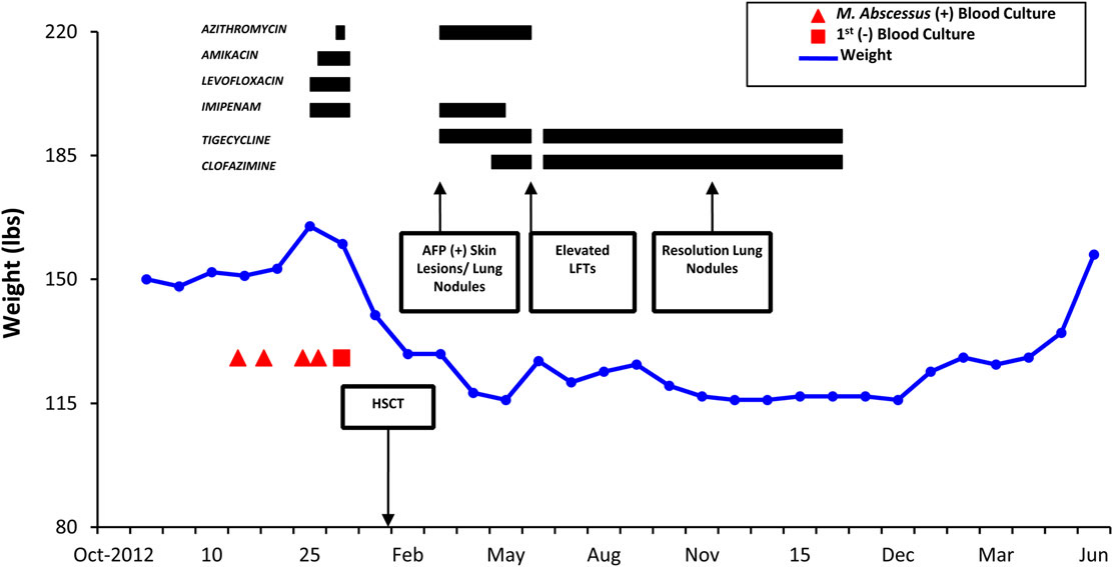
Clinical timeline of events including positive blood cultures, clinical findings, and antimicrobial therapy. Abbreviations: AFP, alpha-fetoprotein; HSCT, hematopoietic stem cell transplantation; LFT, liver function test.

A 68-year-old female patient with a past medical history of diffuse large B-cell (double hit) lymphoma, status post chemotherapy, as well as peripheral stem cell transplantation (PSCT) complicated by disseminated *M. abscessus* infection of the skin was referred to the gastroenterology clinic for evaluation of postprandial abdominal pain, diarrhea, weight loss, and melena. She had been recently started on clofazimine and tigecycline due to previous adverse effects from azithromycin. Two months before PSCT (November 2012), the patient developed a catheter-related blood stream infection with *M. abscessus* that was treated with catheter removal and initial therapy with oral azithromycin, levofloxacin, and amikacin. Two days later, levofloxacin was changed to amikacin after the organism was identified as *M. abscessus* and preliminary susceptibilities determined resistance to fluoroquinolones. The patient completed a 4-week course of antimicrobial therapy with azithromycin, imipenem, and amikacin complicated by amikacin-induced ototoxicity and nephrotoxicity. The patient rapidly cleared the bacteremia within 48 hours after central venous catheter removal and had no other manifestations of infection. In January 2013, she underwent autologous PSCT and within 2 months she developed multiple skin nodules. Nodule biopsies led to the diagnosis of relapsed disseminated *M. abscessus* infection. The patient had previously documented ototoxicity secondary to amikacin and was therefore started on treatment with azithromycin, tigecycline, and imipenem. In addition, the patient was found to have new lung nodules on a thoracic computed tomography scan. After 3 months of therapy (June 2013), she developed hepatotoxicity with severe transaminitis and diarrhea. An extensive hepatology evaluation attributed the hepatotoxicity to azithromycin. Because of the patient's history of ototoxicity and renal injury due to amikacin and the possible hepatotoxicity from azithromycin, clofazimine treatment was initiated. In vitro susceptibility testing of the *M. abscessus* demonstrated susceptibility to tigecycline and clofazimine with resistance to augmentin, bactrim, and linezolid. After obtaining compassionate use and Investigational New Drug (IND) approval through the Florida Department of Health, the patient's regimen was changed to high-dose clofazimine (200 mg by mouth daily) and tigecycline (50 mg IV twice daily). High-dose clofazimine was chosen based on history of disseminated and recurrent mycobacterial infection in a highly immunocompromised patient. Four months later (October 2013), the patient's skin lesions had resolved and repeat chest imaging revealed resolution of the previously noted lung nodules (Figure 1).

One month after initiating clofazimine, the patient developed severe diarrhea with up to 20 watery bowel movements a day. Her diarrhea persisted despite discontinuation of her azithromycin. The evaluation for weight loss demonstrated severe malabsorption including anemia (hemoglobin of 10 g per dL), with macrocytosis (mean corpuscular volume 109.1 fl, red cell distribution width 19.3% [reference range, 11.5%–15.0%]), decreased levels of prealbumin of 8 mg per dL (reference range, 20–40 mg per dL), and decreased albumin (2.1 g per dL [reference range, 3.2–5 .0 g per dL]). Laboratory evaluation also revealed a fat-soluble vitamin deficiency as well as other vitamin deficiencies including folate, vitamin D, 25-OH, and total serum carotene.

After 7 months on high-dose clofazimine, she presented with hematochezia, failure to thrive with a 17-pound weight loss (127 lb to 110 lb), persistent diarrhea, and right lower quadrant abdominal pain. Physical examination revealed tenderness of the right lower quadrant on deep palpation, 2+ pitting edema in the lower extremities bilaterally, and maroon stools on rectal exam. She had no evidence of recurrent mycobacterium infection nor of relapsing lymphoma.

Endoscopic evaluation revealed black pigmentation of the duodenum. Colonoscopy demonstrated severely congested mucosa with petechiae in the terminal ileum (Figure 2A). Small bowel and terminal ileum biopsies revealed intestinal mucosa with focal villous blunting and widening due to numerous crystal-laden macrophages infiltrating the superficial lamina propria, consistent with crystal-storing histiocytosis, which is indicative of clofazimine enteropathy (Figure 2B). Clofazimine and all *M. abscessus* treatment was subsequently discontinued, and the patient had no further gastrointestinal bleeding. She experienced complete resolution of her diarrhea 3 months later, with a 15-pound weight gain noted within 4 months of the discontinuation of the clofazimine. She was initially treated with parenteral nutrition but is now maintained on an oral diet with no recurrence of her gastrointestinal symptoms or of her mycobacterial infection 2 years after the diagnosis of clofazimine enteropathy. This adverse drug effect was reported to the US Food and Drug Administration (FDA).

## DISCUSSION

Clofazimine, a rhimophenazine dye, has both antimicrobial and anti-inflammatory activity. The primary indication for use is multibacillary leprosy, although it has recently been included in the treatment regimen for other mycobacterial infections [4]. Current indications include the treatment of leprosy, discoid lupus erythematosus, disseminated *Mycobacterium avium intracellulare* bacteremia, MDR tuberculosis, and pyoderma gangrenosum. The exact mechanism of action is unknown, but some believe that it may act by increasing the activity of bacterial phospholipase A_2_ resulting in the release of lysophospholipids, enzymatic hydrolysis products, which are toxic to Gram-positive organisms and mycobacteria [1].

New studies describe a pathway for the NADH-dependent redox cycling of the dye resulting in reactive oxygen species production and mycobacterial death [4]. Clofazimine has been reported to induce 3 types of enteropathy: (1) eosinophilic/allergic pattern; (2) Crohn's disease-like pattern with granulomas; and (3) clofazimine-induced crystal-storing histiocytosis [5]. Clofazimine is a lipophilic phenazine dye that, once absorbed, concentrates in lipid-rich tissues. This orally administered medication is primarily absorbed in the reticuloendothelial system, but high concentrations are also found in the breast, liver, and intestines [6]. The medication has an extended half-life of 70 days due to its high degree of lipid solubility. Although clofazimine has shown potential for shortening the duration of tuberculosis treatment, the current dosing is not yet evidence-based and the optimal dosing is unclear [7].

Skin, gastrointestinal tract, and eyes are the sites most commonly affected by clofazimine. Clofazimine may result in cutaneous manifestations of orange-pink discoloration of the skin or, less commonly, ichthyosis. Although these symptoms often resolve after cessation of clofazimine, they rarely warrant discontinuation. The medication is excreted in various body fluids including tears, breast milk, and sputum [8]. Additional side effects include splenic infarction and eosinophilic enteritis.

The most serious side effect of clofazimine, which occurs after several months of therapy with high-dose clofazimine (>100 mg daily) [1, 9], is red crystal deposition in the small bowel lamina propria, which may result in severe and fatal enteropathy [1]. In fact, the intestines are the first organ exposed to high concentrations of the antibiotic. This may result in higher pigmentation in this area with the distal small intestine more heavily involved. Given its lipophilic quality, clofazimine is rapidly absorbed through lymphatic channels and the portal vessels leading to dark pigmentation of lymph nodes as well. Peyer's patches throughout the small intestine would be particularly prone to involvement [10], and perhaps their involvement may be implicated in the development of enteropathy. In one animal study done by Levine and Saltzman [11], clofazimine was administered to rats via a nasogastric tube with 6 doses received over a 2-week period. Microscopic examination of the gastrointestinal tract was later performed, and the reddish discoloration was reported. The colonic mucosa appeared normal, but small intestinal mucosa was hyperpigmented with the distal intestine affected more than the proximal. A granulomatous reaction was noted in both small and large intestinal lymphatic systems, generally located closer to the muscular rather than the mucosal wall. Crystal outlines were also noted within the granulomas. Furthermore, this tissue reaction was more prominent in those rats with higher doses and longer duration of treatment. These effects continued despite discontinuation of the drug [11].

Peyer's patches may have a role in the pathogenesis of clofazimine-induced enteropathy because they protect against foreign antigens and microorganisms in the gut. Peyer's patch involvement due to crystal deposition, as seen with clofazimine, may lead to loss of the protective functions—a phenomenon that may be also involved in Crohn's disease [11]. Furthermore, a study by Baik et al [12] noted the accumulation of clofazimine in the resident macrophages of the lymphatic organs sequestered in intracellular crystal-like drug inclusions in the spleen after the dietary administration of oral clofazimine in mouse models over a 3- to 8-week course. Whether this crystal deposition leads to increased intestinal permeability is yet to be determined.

In summary, this is a unique case of clofazimine-induced crystal storing histiocytosis, presenting as gastrointestinal bleeding and significant malabsorption in an immunocompromised patient with disseminated relapsing *M. abscessus* infection. This most serious side effect of clofazimine, although rare, can result in severe, fatal enteropathy manifesting as severe weight loss, malnutrition, nausea, gastrointestinal bleeding, obstruction, and diarrhea.

Although clofazimine has been in clinical use for over 50 years, it is not widely available in the United States or other countries and requires an IND application from the FDA or compassionate use approval. The “off-label” use of clofazimine is discouraged by the World Health Organization to prevent resistance.

First-line therapy for treatment of *M. abscessus* infections includes a macrolide (azithromycin, clarithromycin) in combination with high-dose cefoxitin or imipenem and low-dose amikacin. Prolonged amikacin use may be contraindicated due to the induction of nephrotoxicity or ototoxicity, particularly in elderly and immunocompromised hosts, and the treatment of these infections are extremely challenging. Unfortunately, *M. abscessus* infections can be resistant to multiple antibiotics, leaving few therapeutic options. For this reason, clofazimine will likely continue to be an acceptable oral agent included in combination therapy regimens for the treatment of MDR *M. abscessus*, particularly for disseminated infections in immunocompromised patients [13, 14].

## CONCLUSIONS

In this era of antimicrobial resistance and opportunistic nontuberculous mycobacteria infections, prescribers should be aware of clofazimine-induced enteropathy, a rare but life-threatening adverse effect previously reported in the leprosy literature. Patients receiving prolonged clofazimine therapy should be monitored for signs and symptoms of enteropathy.
